# Role of sleep on respiratory failure after extubation in the ICU

**DOI:** 10.1186/s13613-021-00863-z

**Published:** 2021-05-08

**Authors:** Arnaud W. Thille, Stephanie Barrau, Clément Beuvon, Damien Marie, Faustine Reynaud, Justine Bardin, Adrien Pépin-Lehalleur, Vanessa Bironneau, Jean-Claude Meurice, Rémi Coudroy, Jean-Pierre Frat, René Robert, Christophe Rault, Xavier Drouot

**Affiliations:** 1 INSERM CIC 1402, ALIVE Research Group, University of Poitiers, Poitiers, France; 2 Médecine Intensive Réanimation, CHU de Poitiers, 2 rue la Milétrie, 86021 Poitiers Cedex, France; 3 Pneumologie, CHU de Poitiers, Poitiers, France; 4 Neurophysiologie clinique et Explorations fonctionnelles, CHU de Poitiers, Poitiers, France

**Keywords:** Airway extubation, Ventilator weaning, Intensive care unit, Sleep, Mechanical ventilation

## Abstract

**Background:**

Sleep had never been assessed immediately after extubation in patients still in the ICU. However, sleep deprivation may alter respiratory function and may promote respiratory failure. We hypothesized that sleep alterations after extubation could be associated with an increased risk of post-extubation respiratory failure and reintubation. We conducted a prospective observational cohort study performed at the medical ICU of the university hospital of Poitiers in France. Patients at high-risk of extubation failure (> 65 years, with any underlying cardiac or lung disease, or intubated > 7 days) were included. Patients intubated less than 24 h, with central nervous or psychiatric disorders, continuous sedation, neuroleptic medication, or uncooperative were excluded. Sleep was assessed by complete polysomnography just following extubation including the night. The main objective was to compare sleep between patients who developed post-extubation respiratory failure or required reintubation and the others.

**Results:**

Over a 3-year period, 52 patients had complete polysomnography among whom 12 (23%) developed post-extubation respiratory failure and 8 (15%) required reintubation. Among them, 10 (19%) had atypical sleep, 15 (29%) had no deep sleep, and 33 (63%) had no rapid eye movement (REM) sleep. Total sleep time was 3.2 h in median [interquartile range, 2.0–4.4] in patients who developed post-extubation respiratory failure vs. 2.0 [1.1–3.8] in those who were successfully extubated (*p* = 0.34). Total sleep time, and durations of deep and REM sleep stages did not differ between patients who required reintubation and the others. Reintubation rates were 21% (7/33) in patients with no REM sleep and 5% (1/19) in patients with REM sleep (difference, − 16% [95% CI − 33% to 6%]; *p* = 0.23).

**Conclusions:**

Sleep assessment by polysomnography after extubation showed a dramatically low total, deep and REM sleep time. Sleep did not differ between patients who were successfully extubated and those who developed post-extubation respiratory failure or required reintubation.

**Supplementary Information:**

The online version contains supplementary material available at 10.1186/s13613-021-00863-z.

## Background

The decision of extubation is a critical moment in the intensive care unit (ICU) because mortality is particularly high in case of extubation failure leading to reintubation [1, 2]. The main reason for reintubation is the occurrence of new respiratory distress which could be due to direct respiratory failure, congestive heart failure, aspiration, inability to clear secretions, or upper airway obstruction [1]. Other causes leading to reintubation include the onset of septic shock, surgical complications and neurological impairment. However, the exact reason explaining reintubation is not always well-understood, and it is sometimes due to multiple factors.

Sleep is an essential physiological activity that is severely altered in ICUs [3–7]. Whereas sleep deprivation may alter inspiratory muscle endurance in healthy subjects [8, 9], sleep abnormalities may alter the ability to breathe spontaneously and precipitate weaning failure in mechanically ventilated patients [10, 11]. Indeed, two recent studies found that altered sleep was associated with prolonged duration of mechanical ventilation and difficult weaning in mechanically ventilated patients [10, 11]. The deep sleep (sleep stage N3) and the rapid eye movement (REM) sleep which are two essential sleep stages that may completely disappear under mechanical ventilation, even in patients who are conscious and not under sedation [6, 10]. In some ICU patients, normal sleep architecture may disappear and be replaced by electroencephalogram (EEG) aspects suggesting atypical sleep recordings that cannot be classified according to the standard criteria [7, 10, 12–14]. It has been shown that patients with atypical sleep under mechanical ventilation had an increased risk of respiratory failure during spontaneous breathing trials, as if brain dysfunction may have an influence on the ability to breathe spontaneously [10, 11]. In patients at high-risk of extubation failure, sleep disturbances might therefore precipitate post-extubation respiratory failure after weaning from the ventilator. However, the role of sleep in extubation outcome in patients ready for extubation after successful spontaneous breathing trial has never previously been studied. Therefore, we hypothesized that sleep alterations immediately after extubation could be associated with an increased risk of post-extubation respiratory failure and reintubation in the ICU.

## Methods

### Study design and patients

We conducted a prospective observational cohort study at a single center between January 2016 and January 2019 at the medical ICU of the university hospital of Poitiers in France. The study was approved by the independent ethics committee of Poitiers (CPP Ouest III) with the registration number 2015-A01726-43, and was registered at http://www.clinicaltrials.gov with the trial registration number NCT02911506. Patients and/or their next of kin were informed and gave their written consent before being included in the study.

Patients were extubated after having successfully undergone a spontaneous breathing trial using T-piece for 1 h [15]. All patients extubated after at least 24 h of mechanical ventilation could be included if they were at high-risk of extubation failure according to the following criteria [16, 17]: older than 65 years of age, with any underlying cardiac or chronic lung disease, or intubated more than 7 days prior to extubation. Patients with intubation less than 24 h, central nervous or psychiatric disease, continuous sedation or neuroleptic medication, or uncooperative, were not included. Other exclusion criteria were minor patient or under protection of the law, patient refusal, decision of do-not-reintubate at time of extubation, or unplanned extubation (accidental or self-extubation).

### Sleep assessment

Sleep was evaluated by complete polysomnography that started in the afternoon following extubation, and was continuously performed until the next morning. Polysomnography recordings were not retained in the final analysis of sleep quality when recording signals were lost before midnight (accidental or voluntary removal). Prophylactic use of noninvasive ventilation to prevent extubation failure was temporarily suspended during the polysomnography so as not to interfere with sleep recordings.

A trained investigator positioned the electrodes which consisted in six EEG channels (F3-A2, F4-A1, C4-A1, C3-A2, O2-A1 and O1-A2) referenced to the contralateral mastoid according to the international 10–20 System for electrode placement [18]. Two electromyograms (chin), and two electro-oculograms were recorded to score REM and non-REM sleep. Sleep recordings were manually scored by a neurologist blinded to the patient’s status. Duration of REM sleep and non-REM sleep stages including light sleep (i.e., sleep stage N1 and N2) and deep sleep (i.e., sleep stage N3) were assessed using the standard criteria of the 2007 American Academy of Sleep Medicine [19]. Atypical sleep was detected by the absence of stage-N2 markers (absence of K complexes and sleep spindles) as previously reported [10, 14]. Atypical sleep is a form of sleep frequently observed in ICU patients, which makes it no longer possible to identify the 3 usual non-REM sleep stages according to the standard criteria. However, REM sleep can still be detected and it remains possible to differentiate sleep state from wakefulness state and to thereby quantify sleep duration. EMG and EOG are more active during wake state than during sleep while decreased EMG activity with specific eye movements occurs during REM sleep. To be able to differentiate atypical sleep from pathological wakefulness characterized by excessive slow wave activity, an eye-opening test was systematically performed by the neurologist investigator (XD) at the beginning of polysomnography in order to assess the EEG frequency in wake state. EEG reactivity was assessed according to EEG rhythm on an O2-A1 electrode [14]. Immediate disappearance or frank attenuation of the background EEG rhythm at eye-opening, which was replaced by fast low-amplitude frequencies and maintained as long as the eyes were open, was considered as normal EEG reactivity. Moderate and brief attenuation was considered as altered EEG reactivity. An undetectable or very small difference between EEG patterns with the eyes closed and the eyes open was considered as no EEG reactivity.

Sleep fragmentation was assessed using the number of arousals and awakenings per hour of sleep. Noise being one of the most sleep-disruptive factors, the average sound pressure level (expressed in decibels) was recorded with a microphone placed above the patients’ heads throughout the polysomnography [20, 21].

### Clinical assessment

Strength of limb muscles was measured at the time of polysomnography using the Medical Research Council (MRC) sum-score and ICU-acquired paresis was defined as a MRC sum-score below 48 points [22]. Strength of inspiratory muscles and central respiratory drive were assessed with a portable monitor (FluxMed GrH, MBMED, Buenos Aires, Argentina) by measuring maximal inspiratory pressure and negative airway pressure generated against occlusion during the first 0.1 s of spontaneous inspiration (*P*_0.1_) [23, 24].

Neurological function was clinically assessed at the time of polysomnography by measuring the Richmond Agitation–Sedation Scale (RASS) for consciousness [25] and the Intensive Care Delirium Screening Checklist (ICDSC) for delirium [26]. Altered consciousness was defined as RASS < 0 and delirium as ICDSC ≥ 4.

### Outcomes

The main outcomes were sleep quantity including total sleep time and durations of light, deep and REM sleep stages, and sleep quality including the proportion of patients with atypical sleep or no REM sleep [10]. Sleep quantity and quality were assessed among all extubated patients, in patients who developed post-extubation respiratory failure, in patients who required reintubation, and in those who were successfully extubated.

Post-extubation respiratory failure was defined by the presence of at least two criteria among the following: a respiratory rate above 25 breaths per minute, clinical signs suggesting respiratory distress, respiratory acidosis defined as pH below 7.35 units and PaCO_2_ above 45 mmHg, hypoxemia defined as a need for FiO_2_ at least 50% to maintain SpO_2_ level at least 92% or a PaO_2_/FiO_2_ ratio ≤ 150 mm Hg. Patients were immediately reintubated in case of cardiac or respiratory arrest, neurological failure (altered consciousness with a Glasgow Coma Scale below 12), or severe respiratory failure defined by the presence of at least two criteria among the following: respiratory rate above 35 breaths per minute, clinical signs suggesting respiratory distress with activation of accessory respiratory muscles, respiratory acidosis defined as pH below 7.25 units and PaCO_2_ above 45 mmHg, hypoxemia defined as a need for FiO_2_ at 80% or more to maintain SpO_2_ level at 92% or more or a PaO_2_/FiO_2_ ratio ≤ 100 mmHg.

### Statistical analysis

We hypothesized that patients with poor sleep quality (with atypical sleep or no REM sleep) may have reintubation rates reaching 40%, i.e., close to the reintubation rates reported in the most severe patients with severe cardiac heart failure, severe limb weakness or ineffective cough [17, 27, 28], versus only 10% in patients with good sleep quality, i.e., as patients at low-risk of reintubation [29]. Enrollment of 64 patients with adequate polysomnography was determined to provide a power of 80% and to show an absolute difference of 30% in rate of reintubation between patients with poor sleep quality and those with good sleep quality.

Continuous variables were expressed as median and interquartile range [IQR 25th–75th percentiles], and qualitative variables were expressed as number and percentage. Patients who had poor sleep quality (atypical sleep or no REM) were compared to patients with normal sleep architecture. Kaplan–Meier curves were plotted to assess the time from polysomnography to reintubation and were compared by means of the log-rank test. Then, patients who developed post-extubation respiratory failure and those who required reintubation up until ICU discharge were compared to the others. All variables were compared using the non-parametric Fisher exact test for categorical variables and the Mann–Whitney test for continuous variables. As sedation may influence sleep, we performed a non-parametric Spearman correlation between these 2 variables. A two-tailed *P*-value of less than 0.05 was considered as statistically significant. All analyses were performed using R statistical package (online at http://www.R-project.org).

## Results

**Fig. 1:**
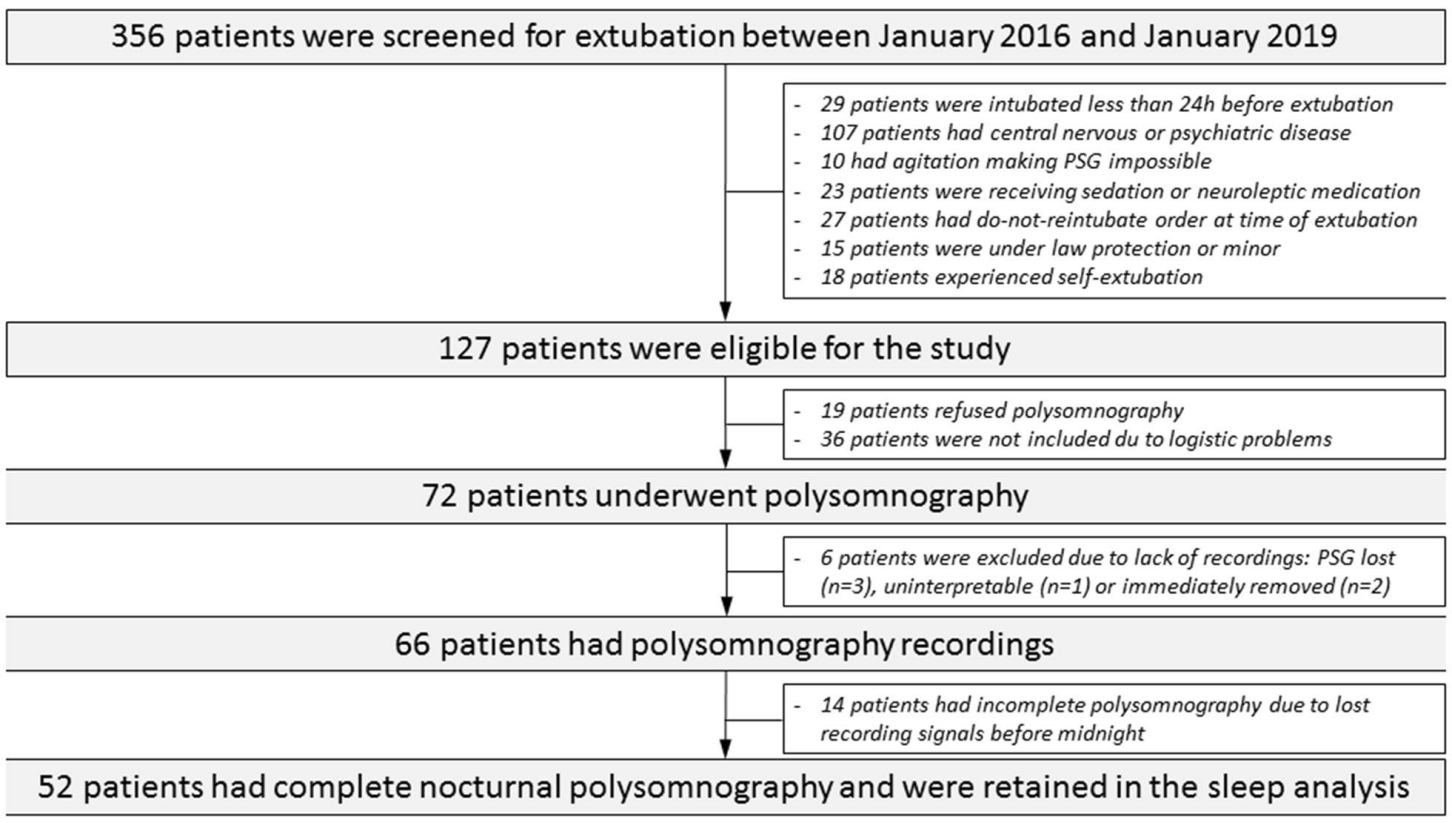
Flowchart of the patients

Over a 3-year period, 66 patients had adequate polysomnography recordings (Fig. 1). Among them, polysomnography was complete during the night in 52 patients (79%), whereas electrodes were accidentally or voluntarily removed, and polysomnography recordings were consequently lost before midnight in 14 patients (21%). As compared to patients who had complete polysomnography during the night, patients in whom polysomnography recording were lost before midnight were more likely to be male (93% vs. 67%, *p* = 0.04) and had a higher delirium score: 2.0 [1.3–3.0] vs. 0.0 [0.0–1.5], *p* = 0.03.

### Characteristics of the 52 patients who had complete polysomnography during the night

Among these 52 patients, 12 patients (23%) developed post-extubation respiratory failure and 8 (15%) required reintubation in the ICU, all for respiratory failure, with a median interval of 2 days [IQR 0–3] after extubation and only 1 day [IQR 0–2] after disconnecting polysomnography. Polysomnography was performed the first night following extubation in 50 patients (96%), and the second night in 2 patients (4%). Midazolam had been used as sedation in 49 patients (94%) for 3 days in median ([IQR 1–8] with a cumulative dose of 360 mg in median [IQR 140–1545]). At time of polysomnography, the number of sedation-free days was 3 days in median [IQR 2–5] and the level of consciousness was the following: 1 patient was drowsy (RASS − 1), 48 patients (92%) were alert and calm (RASS 0), 2 patients were restless (RASS + 1), and 1 patient was agitated (RASS + 2). The proportion of patients with delirium was 15% (8 patients). When adding two more patients who developed delirium after polysomnography, the rate reached 19%.

### Sleep analysis among the 52 patients who had complete polysomnography during the night

Total sleep time was only 2.4 h [IQR 1.1–4.2] (Table 1). Median deep sleep duration was 17 min [IQR 0–66], representing 11% of total sleep time [IQR 0–39], and median REM sleep duration was 0 min [IQR 0–8], representing 0% of total sleep time [IQR 0–3]. Among these 52 patients, 10 (19%) had atypical sleep, 15 (29%) had no deep sleep, and 33 (63%) had no REM sleep. Only 15 patients (29%) had normal sleep architecture, whereas the 37 other patients had poor sleep quality (i.e., with atypical sleep or complete absence of REM sleep). As compared to patients with normal sleep architecture, those with poor sleep quality had lower sleep quantity and especially lower deep sleep, and lower negative airway pressure generated against occlusion during the first 0.1 s of spontaneous inspiration (*P*_0.1_) indicating altered central respiratory drive (Table 2).

**Table 1:** Characteristics, sleep quality and outcomes of the 52 patients included in the study

	*N* = 52
Patient characteristics	
Age (years)	68 [59–76]
Male sex, *n* (%)	35 (67%)
Body mass index (kg/m^2^)	30 [25–34]
Simplified Acute Physiological Score II at admission (points)	48 [36–62]
Acute respiratory failure as main reason of intubation, *n* (%)	43 (83%)
Duration of mechanical ventilation before extubation (days)	9 [4–16]
Sedation before polysomnography (number of days)	3 [2–9]
Sedation-free days at time of polysomnography (days)	3 [2–5]
Difficult or prolonged weaning^a^, *n* (%)	18 (35%)
Respiratory parameters before extubation	
Maximal inspiratory pressure (cmH_2_O)	51 [36–62]
*P*_0.1_ (cmH_2_O)	3.4 [3.3–3.6]
pH, units	7.46 [7.43–7.49]
PCO_2_ (mmHg)	41[36–45]
PaO_2_/FiO_2_ (mmHg)	250 [186–300]
Ineffective cough, *n* (%)	6 (12%)
Abundant secretions, *n* (%)	11 (21%)
Clinical parameters at time of PSG	
Sequential Organ Failure Assessment (points)	3 [2–4]
Richmond Agitation Sedation Scale (points)	0 [0–0]
Intensive care delirium screening checklist (points)	0.0 [0.0–1.5]
Delirium, *n*/*n* assessed (%)	8/51 (16%)
Medical Research Council (MRC) score (points)	52 [36–60]
ICU-acquired weakness (MRC < 48), *n*/*n* total (%)	17/44 (39%)
Prophylactic noninvasive ventilation, *n* (%)	26 (50%)
Sleep quantity	
Duration of polysomnography recording (h)	16.7 [14.9–17.4]
Sleep duration (h)	2.4 [1.1–4.2]
Sleep efficiency (%)	16 [7–24]
Duration of light or atypical sleep (min)	94 [46–175]
Duration of deep sleep stage 3 (min)	17 [0–66]
Duration of REM sleep stage (min)	0 [0–8]
Sleep quality	
Absence of deep sleep, *n* (%)	15 (29%)
Absence of REM sleep, *n* (%)	33 (63%)
Atypical sleep, *n* (%)	10 (19%)
Pathological wakefulness, *n* (%)	12 (23%)
Altered EEG reactivity at eye-opening test, *n*/*n* assessed (%)	15/48 (31%)
Sleep fragmentation	
Arousals and awakenings, events per hour of sleep	25 [13–32]
Average sound pressure level, decibels	57 [55–61]
Sound level events above 60 decibels, *n* events/h	27 [19–39]
Outcomes	
Post-extubation respiratory failure, *n* (%)	12 (23%)
Reintubation within the 7 days following extubation, *n* (%)	7 (13%)
Reintubation in ICU, *n* (%)	8 (15%)
Mortality in ICU, *n* (%)	1 (2%)

Values are given in median [25th–75th percentiles] and compared using the non-parametric Fisher exact test for categorical variables and the Mann–Whitney test for continuous variables

*P*_0.1_: negative airway pressure generated against occlusion during the first 0.1 s of spontaneous ventilation; REM sleep: rapid eye movement sleep; ICU: intensive care unit; EEG reactivity: electroencephalographic reactivity

^a^Difficult or prolonged weaning refer to patients who were not extubated within the first 24 h after the initial spontaneous breathing trial

**Table 2:** Comparison of patients with normal sleep architecture and those with atypical sleep or no rapid eye movement (REM) sleep

	Normal sleep (*N* = 15)	Atypical or no REM sleep (*N* = 37)	*P* value
Patient characteristics			
Age (years)	71 [61–76]	67 [58–76]	0.34
Male sex, *n* (%)	11 (73%)	24 (65%)	0.75
Body mass index (kg/m^2^)	31 [27–37]	28 [25–32]	0.19
Simplified Acute Physiological Score II at admission (points)	40 [30–57]	50 [38–66]	0.13
Duration of mechanical ventilation before extubation (days)	4 [3–15]	9 [6–16]	0.14
Sedation before polysomnography (number of days)	2 [1–6]	4 [2–9]	0.09
Sedation-free days at time of polysomnography (days)	2 [2–6]	3 [2–5]	0.29
Difficult or prolonged weaning^a^, *n* (%)	6 (40%)	12 (32%)	0.75
Respiratory parameters before extubation			
Maximal inspiratory pressure (cmH_2_O)	55 [37–60]	50 [36–62]	0.73
*P*_0.1_ (cmH_2_O)	4 [3, 4]	3 [3, 4]	*0.04*
pH, units	7.45 [7.44–7.49]	7.46 [7.43–7.49]	0.96
PCO_2_ (mmHg)	41 [39–47]	41 [34–44]	0.11
PaO_2_/FiO_2_ (mmHg)	200 [183–276]	256 [195–303]	0.24
Ineffective cough, *n* (%)	1 (7%)	5 (14%)	0.66
Abundant secretions, *n* (%)	4 (27%)	7 (19%)	0.71
Clinical parameters at time of PSG			
Sequential Organ Failure Assessment (points)	3.0 [2.0–3.5]	3.0 [2.0–4.0]	0.63
Richmond Agitation Sedation Scale (points)	0 [0–0]	0 [0–0]	0.53
Intensive care delirium screening checklist (points)	0 [0–0]	1 [0–2]	0.086
Delirium, *n*/*n* assessed (%)	2/14 (14%)	6 (16%)	> 0.99
Medical Research Council (MRC) score (points)	60 [57–60]	48 [33–59]	*0.01*
ICU-acquired weakness (MRC < 48), *n*/*n* total (%)	2/12 (17%)	15/32 (47%)	0.09
Prophylactic noninvasive ventilation, *n* (%)	10 (67%)	16 (43%)	0.22
Sleep quantity			
Duration of polysomnography recording (h)	16.9 [15.5–17.5]	16.2 [14.2–17.2]	0.27
Sleep duration (h)	4.2 [3.5–5.0]	1.7 [0.9–3.4]	*< 0.001*
Sleep efficiency (%)	25 [21–30]	11 [6–18]	*< 0.001*
Duration of light or atypical sleep (min)	129 [70–214]	87 [44–123]	0.12
Duration of deep sleep stage 3 (min)	99 [40–120]	4 [0–36]	*< 0.001*
Duration of REM sleep stage (min)	13 [10–24]	0 [0–0]	*< 0.001*
Sleep quality			
Absence of deep sleep, *n* (%)	0 (0%)	15 (41%)	*0.002*
Absence of REM sleep, *n* (%)	0 (0%)	33 (89%)	*< 0.001*
Atypical sleep, *n* (%)	0 (0%)	10 (27%)	*0.046*
Pathological wakefulness, *n* (%)	1 (7%)	11 (30%)	0.14
Altered EEG reactivity at eye-opening test, n/n assessed (%)	3/14 (21%)	12/34 (35%)	0.50
Sleep fragmentation			
Arousals and awakenings, events per hour of sleep	37 [22–40]	42 [24–50]	0.44
Average sound pressure level, decibels	58 [55–64]	57 [55–59]	0.47
Sound level events above 60 decibels, *n* events/h	25 [18–41]	28 [20–37]	0.85
Outcomes			
Post-extubation respiratory failure, *n* (%)	2 (13%)	10 (27%)	0.47
Reintubation in ICU, *n* (%)	1 (7%)	7 (19%)	0.41
Mortality in ICU, *n* (%)	0 (0%)	1 (3%)	> 0.99

Values are given in median [25th–75th percentiles] and compared using the non-parametric Fisher exact test for categorical variables and the Mann–Whitney test for continuous variables

*P* values are indicated in italic when the variables are statistically different between the 2 groups (*P* < 0.05)

*P*_0.1_: negative airway pressure generated against occlusion during the first 0.1 s of spontaneous ventilation; REM sleep: rapid eye movement sleep; ICU: intensive care unit; EEG reactivity: electroencephalographic reactivity

^a^Difficult or prolonged weaning refer to patients who were not extubated within the first 24 h after the initial spontaneous breathing trial

**Fig. 2:**
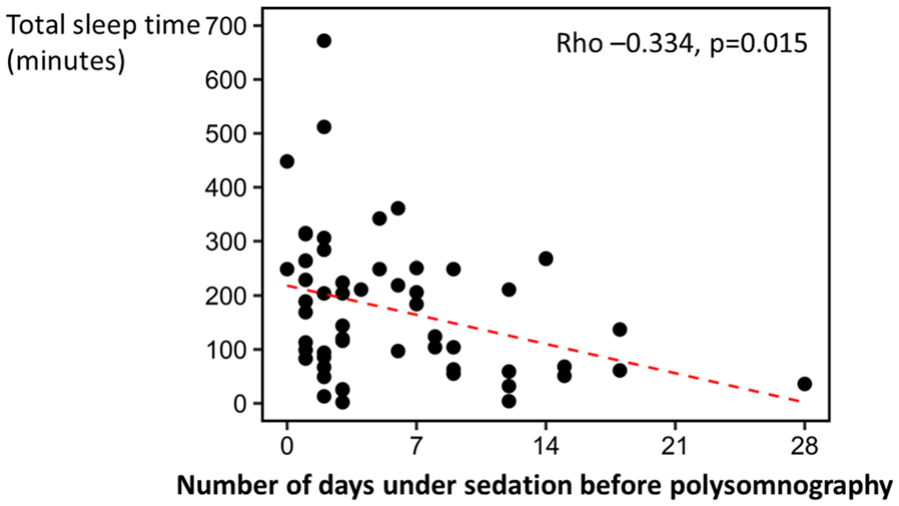
The duration of sedation before polysomnography (*X* axis) and total sleep time (*Y* axis) were significantly correlated (Rho − 0.334, *p* = 0.015) meaning that the longer the duration of sedation before polysomnography, the shorter the total sleep time

The duration of sedation before polysomnography and total sleep time were significantly correlated (Rho − 0.334, *p* = 0.02), meaning that the longer the duration of sedation before polysomnography the shorter the total sleep time (Fig. 2). Patients with no REM sleep had received sedation for a longer duration before polysomnography than patients with REM sleep (6 days in median [IQR 2–10] vs. 2 [IQR 1–6], *p* = 0.03).

### Comparison between patients who developed post-extubation respiratory failure or required reintubation and the others

**Fig. 3:**
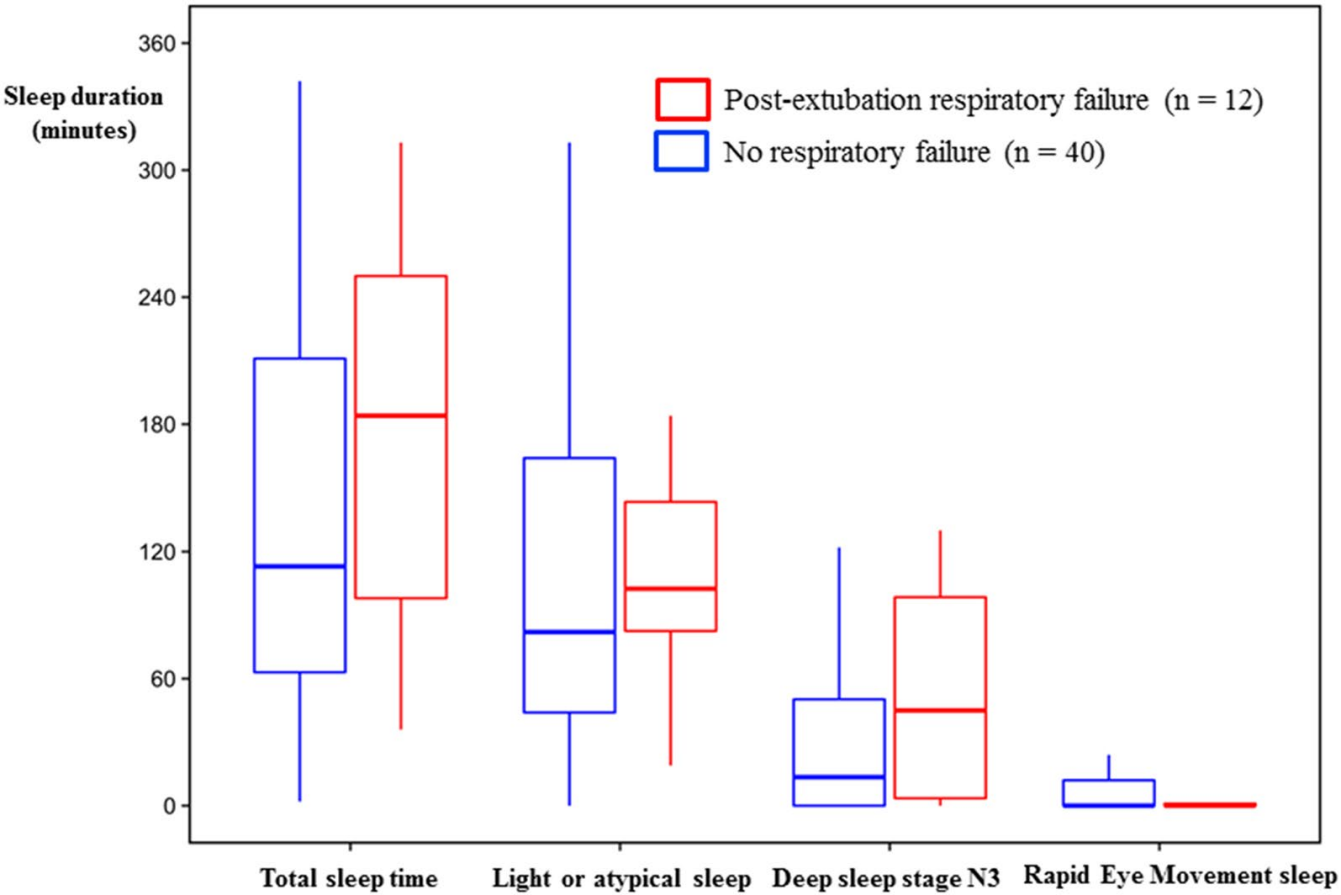
Box plots showing median duration and interquartile range [IQR 25th–75th percentiles] of total sleep, light or atypical sleep, deep sleep stage 3 and rapid eye movement (REM) sleep stage. No significant differences were found between patients who developed post-extubation respiratory failure (red bars) and those who were successfully extubated (blue bars)

Comparison between patients who developed post-extubation respiratory failure and those with no respiratory failure is given in Table 3. Patients who developed post-extubation respiratory failure had lower maximal inspiratory pressure before extubation than the others. Total sleep time and durations of deep and REM stages of sleep did not differ between patients who developed post-extubation respiratory failure and those who were successfully extubated with no respiratory failure (Fig. 3).

**Table 3:** Comparison of patients who developed post-extubation respiratory failure and those who succeeded extubation with no respiratory failure

	No respiratory failure (*N* = 40)	Post-extubation respiratory failure (*N* = 12)	*P *value
Patient characteristics			
Age (years)	68 [59–76]	65 [58–72]	0.52
Male sex, *n* (%)	27 (67%)	8 (67%)	0.99
Body mass index (kg/m^2^)	30 [25–33]	30 [25–37]	0.47
Simplified Acute Physiological Score II at admission (points)	48 [36–62]	51 [37–59]	0.94
Duration of mechanical ventilation before extubation (days)	8 [4–16]	10 [8–16]	0.36
Sedation before polysomnography (*n* days)	3 [2–8]	7 [2–11]	0.35
Sedation-free days at time of polysomnography (*n* days)	3 [2–5]	4 [1–6]	0.96
Difficult or prolonged weaning, *n* (%)	16 (40%)	2 (17%)	0.18
Respiratory parameters before extubation			
Maximal inspiratory pressure (cmH_2_O)	56 [37–64]	34 [29–50]	*0.02*
*P*_0.1_ (cmH_2_O)	3 [3, 4]	3 [3, 4]	0.56
pH	7.45 [7.43–7.48]	7.48 [7.47–7.50]	0.10
PCO_2_ (mmHg)	41 [36–46]	40 [35–41]	0.16
PaO_2_/FiO_2_ (mmHg)	253 [197–314]	244 [185–262]	0.34
Ineffective cough, *n* (%)	3 (7%)	3 (25%)	0.13
Abundant secretions, *n* (%)	9 (22%)	2 (17%)	0.99
Clinical parameters at time of PSG			
Sequential Organ Failure Assessment (points)	3 [2–4]	4 [2–5]	0.12
Richmond Agitation Sedation Scale (points)	0 [0–0]	0 [0–0]	0.79
Intensive care delirium screening checklist (points)	0 [0–1]	1 [0–4]	0.26
Delirium, *n*/*n* assessed (%)	4/39 (10%)	4/12 (33%)	0.08
Medical Research Council (MRC) score (points)	54 [37–60]	45 [29–60]	0.47
ICU-acquired weakness (MRC < 48), *n*/*n* total (%)	11/34 (32%)	6/10 (60%)	0.15
Prophylactic noninvasive ventilation, *n* (%)	20 (50%)	6 (50%)	0.99
Sleep quantity			
Duration of polysomnography recording (h)	16.4 [14.6–17.3]	16.8 [15.8–17.4]	0.71
Sleep duration (h)	2.0 [1.1–3.8]	3.2 [2.0–4.4]	0.34
Sleep efficiency (%)	15 [7–23]	20 [11–27]	0.32
Duration of light or atypical sleep, min	83 [44–177]	103 [83–144]	0.53
Duration of deep sleep stage 3 (min)	14 [0–50]	45 [4–99]	0.16
Duration of REM sleep stage, min	0 [0–12]	0 [0–1]	0.42
Sleep quality			
Absence of deep sleep, *n* (%)	24 (60%)	9 (75%)	0.50
Absence of REM sleep, *n* (%)	13 (33%)	2 (17%)	0.47
Atypical sleep, *n* (%)	8 (20%)	2 (17%)	0.99
Pathological wakefulness, *n* (%)	10 (25%)	2 (17%)	0.71
Altered EEG reactivity at eye-opening test, *n*/*n* assessed (%)	12/37 (32%)	3/11 (27%)	0.99
Sleep fragmentation			
Arousals and awakenings, events per hour of sleep	27 [16–36]	21 [13–28]	0.42
Average sound pressure level, decibels	56 [54–59]	60 [57–52]	0.19
Sound level events above 60 decibels, *n* events/h	25 [18–34]	39 [29–39]	0.19

Values are given in median [25th–75th percentiles] and compared using the non-parametric Fisher exact test for categorical variables and the Mann–Whitney test for continuous variables

*P* values are indicated in italic when the variables are statistically different between the 2 groups (*P* < 0.05)

*P*_0.1_: negative airway pressure generated against occlusion during the first 0.1 s of spontaneous ventilation; REM sleep: rapid eye movement sleep; ICU: intensive care unit; EEG reactivity: electroencephalographic reactivity

^a^Difficult or prolonged weaning refer to patients who were not extubated within the first 24 h after the initial spontaneous breathing trial

**Fig. 4:**
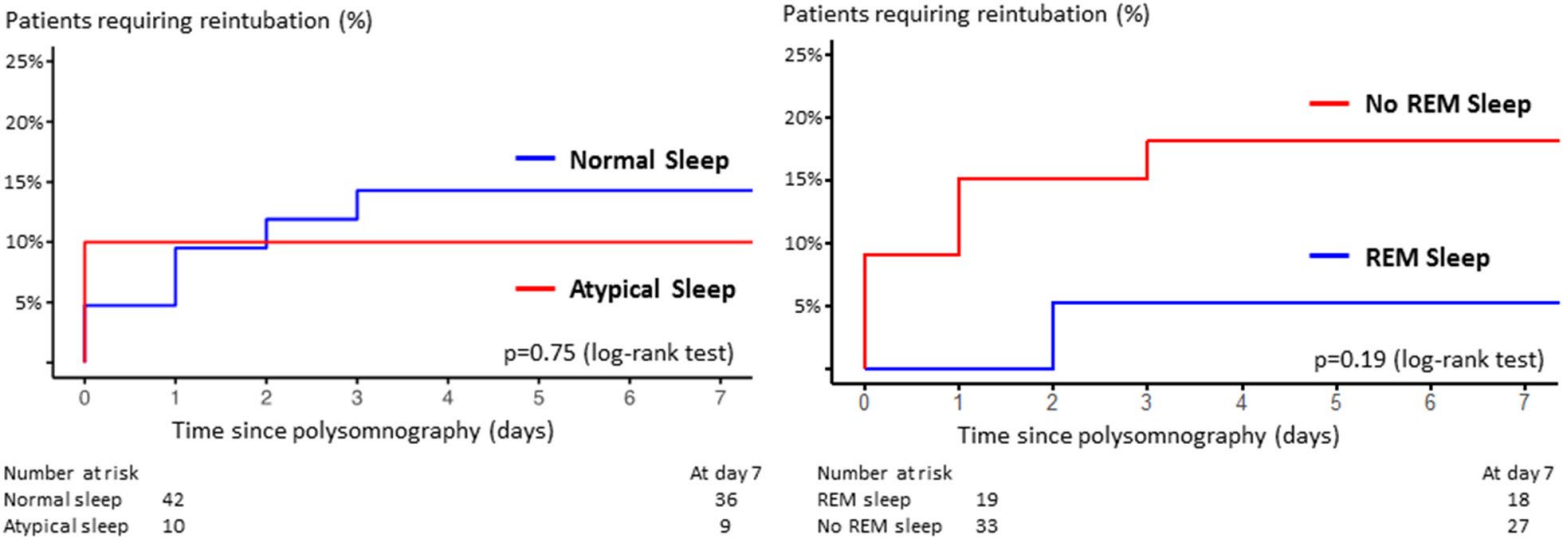
Kaplan–Meier curves showing time from polysomnography to reintubation according to the presence of atypical sleep or normal sleep (at left) and the presence or not of rapid eye movement (REM) sleep. The rates of reintubation within the first 7 days after polysomnography was 14% (6/42) in patients with normal sleep vs. 10% (1/10) in those with atypical sleep (p = 0.75 using log-rank test), and was 5% (1/19) in patients with REM sleep vs. 18% (6/33) in those with no REM sleep (*p* = 0.19 using log-rank test)

Comparison between patients who required reintubation in the ICU and those who were successfully extubated is given in Additional file 1: Table S1. Patients who required reintubation had spent more time under sedation and were more likely to exhibit ICU-acquired weakness with a lower MRC sum-score than the others. Total sleep time and durations of deep and REM sleep stages did not differ between patients who required reintubation and those who were successfully extubated. Among the 8 patients who required reintubation, all except one had complete absence of REM during polysomnography. Reintubation rates were 5% (1/19) in patients with REM sleep and 21% (7/33) in patients with no REM sleep (difference, − 16% [95% CI − 33% to 6%]; *p* = 0.23) (Fig. 4). One patient with no REM sleep was reintubated beyond the first 7 days after polysomnography.

## Discussion

In this prospective physiological study assessing sleep after extubation, total sleep time and durations of deep and REM sleep stages were dramatically low. Nearly two-thirds of patients had complete absence of REM sleep. However, sleep did not seem to have an influence on the risk of post-extubation respiratory failure or reintubation in the ICU.

### Sleep quantity in ICU patients after extubation

Sleep alterations are particularly frequent in the ICU with reduction or complete absence of deep and REM sleep stages even in patients conscious and not under sedation [6, 10, 11]. In previous studies, median total sleep time ranged between 4 and 8 h in patients under mechanical ventilation [6, 10, 11]. Total sleep time observed in the present study was even more reduced (2.4 h in median), although patients were conscious, breathing spontaneously and had been free from sedation for several days. To our knowledge, sleep had never previously been assessed immediately after extubation in patients still in the ICU. By contrast, several studies have assessed sleep quantity within the first days after ICU discharge [30, 31]. In these studies, total sleep time was markedly longer than in our study, exceeding 4 h in one study [31] and close to normal (around 10 h) in the second one [30], whereas REM sleep was preserved in more than 90% of the cases [30, 31]. Our study suggests that immediately after extubation sleep could be the worst in terms of quantity and quality. During REM sleep, accessory inspiratory muscles are inhibited and the diaphragm remains the only active inspiratory muscle [32]. Consequently, this sleep stage may lead to hypoxemic episodes and precipitate respiratory failure [33]. Complete absence of REM sleep was particularly frequent in our patients (63%). Whether the dramatically reduced quantity of sleep and complete absence of REM is a protective mechanism to avoid respiratory failure or simply insomnia due to anxiety following ventilator separation needs future investigations.

### Influence of sleep on weaning from mechanical ventilation and extubation

Two recent studies showed that patients with altered sleep during the weaning period were more likely to exhibit prolonged weaning and delayed extubation [10, 11]. In the first study, atypical sleep was particularly frequent (44%) and was associated with more prolonged weaning [10]. However, patients with atypical sleep did not exhibit more altered respiratory muscle strength, suggesting that brain dysfunction may precipitate respiratory failure and alter ability to breathe spontaneously. In line with these findings, it has been shown that in healthy subjects sleep deprivation may reduce respiratory endurance by altering cortical respiratory command [8, 9]. In the second study, Dres et al. have shown that pathological wakefulness (observed in 38% of patients) may be associated with delayed extubation [11]. EEG abnormalities such as atypical sleep or pathological wakefulness seem particularly frequent under mechanical ventilation and maybe favored by more prolonged sedation. In the present study, the duration of sedation was significantly correlated with total sleep time meaning that the longer the duration of sedation received under mechanical ventilation, the shorter the duration of sleep after extubation. The proportion of patients with atypical sleep or pathological wakefulness was lower (around 20%) than in previous studies including patients under mechanical ventilation, and this may be explained by the fact that patients were studied later after sedation withdrawal. By contrast, we observed no difference of sleep quantity between patients who developed post-extubation respiratory failure and those with no respiratory failure. Sleep durations tended even to be shorter in patients who were successfully extubated, as if staying awake could prevent respiratory failure. Although patients with altered sleep had lower central respiratory drive (as indicated by lower *P*_0.1_), their risk of post-extubation respiratory failure or reintubation was not significantly increased as compared to those with normal sleep. Once a trial is successful and the patient able to breathe spontaneously, sleep quality may no longer be a determinant for extubation failure, which may be more likely be due to multiple factors including underlying cardiac or respiratory disease, cough strength, ICU-acquired weakness and respiratory muscle dysfunction [17, 28, 34]. However, we cannot rule out the possibility that the sleep times observed in our study after extubation were so short in the overall population that the potential impact on prognosis of altered sleep cannot be accurately assessed. As a consequence, it was not possible to assess whether highly restorative sleep may have beneficial effects on extubation outcome. Moreover, whereas sleep did not seem to have an impact on short-term outcome, especially with regard to the risk of post-extubation respiratory failure or of reintubation, sleep is an essential physiological activity permitting physical and neurobehavioral restoration, and could be of major importance in long-term rehabilitation of critically ill patients.

## Limitations

The main limitation of the study is the relatively small sample of patients studied by polysomnography. Therefore, the sample size could have been underpowered to detect a difference between the 2 groups. Knowing that reintubation rates are only around of 15% in overall population [1], we planned to observe around 10 events. While polysomnography is the reference method to assess sleep, it remains a heavy technique to implement in the ICU, and it is difficult to carry out polysomnography in large cohorts of critically ill patients. Not all patients could be screened over the 3-year study period, and inclusions could only be done over discontinuous periods when all investigators were available. However, previous studies, in which the number of patients included was even lower than in our study, showed that altered sleep may have be associated with difficult weaning and may delay extubation [10, 11]. In physiological studies, a major effect may be detected with a relatively small sample of patients. Therefore, although we cannot definitely rule out the possible role of the deleterious effects of poor sleep on post-extubation respiratory failure, this role is likely to be only minimal and, in any case, weaker than during the weaning period under mechanical ventilation.

Another major limitation is that the study was performed in a single center. Consequently, the dramatically low duration of sleep recorded in the present study may be due to an environment not conducive to sleep in that unit. However, a previous study performed in the same unit during the weaning period of mechanically ventilated patients found markedly longer sleep quantity (4.6 h in median) than in the present study [10]. Although the number of arousals per hour of sleep may appear particularly high in our unit, sleep fragmentation to this extent is frequently reported in other ICUs [11, 35–38]. Concerning noise, average sound pressure levels around 55–60 decibels are close to the usual levels reported in ICUs and much less than in other units [20, 39].

A recent large clinical trial has showed that noninvasive ventilation may prevent post-extubation respiratory failure and reintubation in patients at high-risk of extubation failure such as those we have included in the present study [40]. All patients have not been treated with noninvasive ventilation immediately after extubation, and that could have influenced the risk of extubation failure.

Lastly, the vast majority of patients had received benzodiazepines during their ICU stay, a sedation that may alter sleep quality. Clinical practice guidelines recommend using either propofol or dexmedetomidine over benzodiazepines for sedation in ICU patients [41]. However, the strength of this recommendation was only conditional given the low certainty of evidence. Shortly after these guidelines were issued, a large-scale randomized controlled trial including 4000 mechanically ventilated patients in ICUs showed that early use of dexmedetomidine was associated with an increased risk of death compared with usual care, and that benzodiazepines were widely still used [42]. Whereas benzodiazepines may lengthen mechanical ventilation duration as compared with other sedatives, no clinical trial showed that benzodiazepines were associated with increased sleep alterations in ICU [41]. Dexmedetomidine may lengthen total sleep quantity, but seems not to improve deep and REM sleep stages [43, 44]. Propofol suppresses REM sleep and is therefore not an alternative medication to improve sleep in ICU patients [45]. Consequently, we believe our findings on sleep analysis might be generalized outside of our center, even in units that would use other sedatives.

Visual analysis of sleep stages according to the standard criteria is particularly difficult in ICU patients who have received sedatives, and these EEG abnormalities could be due to brain dysfunction rather than sleep disturbances. Automatic detection tools of these EEG abnormalities could facilitate the identification of pathological wakefulness, atypical sleep, or even different sleep stages [11].

## Conclusions

Sleep recordings by polysomnography immediately after extubation in mechanically ventilated patients were characterized by a dramatically low duration of total sleep time and deep sleep, and a particularly large proportion of patients with no REM sleep. Sleep quantity and quality did not differ between patients who developed post-extubation respiratory failure or who were reintubated and those who were successfully extubated.

## Supplementary Information

**Additional file 1: Table S1.** Comparison of patients who required reintubation in the ICU and those who succeeded extubation.

## Data Availability

The datasets used and/or analyzed during the current study are available from the corresponding author on reasonable request.
