# Supplementary material for: Role of sleep on respiratory failure after extubation in the ICU
Source: Ann Intensive Care. 2021 May 8;11:71. doi: 10.1186/s13613-021-00863-z (PMC8105690; doi:10.1186/s13613-021-00863-z)
Supplement: Supplementary file 1 — Additional file 1: Table S1. Comparison of patients who required reintubation in the ICU and those who succeeded extubation. [file 13613_2021_863_MOESM1_ESM.docx]

**Table S1:** Comparison of patients who required reintubation in the ICU and those who succeeded extubation

|  | **Extubation success**  **(N = 44)** | **Reintubation**  **(N = 8)** | ***P***  **value** |
| --- | --- | --- | --- |
| ***Patient characteristics*** |  |  |  |
| Age (years) | 69 [59-76] | 61 [58-65] | 0.205 |
| Male sex, n (%) | 30 (68%) | 5 (63%) | 0.999 |
| Body mass index (kg/m^2^) | 30 [26-35] | 26 [24-29] | 0.119 |
| Simplified acute physiological score II at admission (points) | 49 [36-60] | 41 [31-65] | 0.704 |
| Duration of mechanical ventilation before extubation (days) | 3 [2-7] | 9 [5-15] | **0.043** |
| Sedation before polysomnography (number of days) | 3 [2-5] | 3 [1-5] | 0.389 |
| Sedation-free days at time of polysomnography (days) | 16 (36%) | 2 (25%) | 0.698 |
| ***Respiratory parameters before extubation*** |  |  |  |

| Maximal inspiratory pressure (cm H_2_O) | 55 [36-64] | 49 [29-49] | 0.069 |
| --- | --- | --- | --- |

| P_0.1_ (cm H_2_O) | 3 [3-4] | 3 [3-4] | 0.171 |
| --- | --- | --- | --- |
| pH | 7.46 [7.43-7.49] | 7.48 [7.43-7.49] | 0.832 |
| PCO_2_ (mm Hg) | 41 [37-45] | 37 [31-42] | 0.104 |
| PaO_2_/FiO_2_ (mm Hg) | 250 [185-310] | 251 [227-253] | 0.925 |
| Ineffective cough, n (%) | 4 (9%) | 2 (25%) | 0.227 |
| Abundant secretions, n (%) | 10 (23%) | 1 (12%) | 0.671 |
| ***Clinical parameters at time of PSG*** |  |  |  |
| Sequential organ failure assessment (points) | 3 [2-4] | 3 [2-5] | 0.919 |
| Richmond agitation sedation scale (points) | 0 [0-0] | 0 [0-0] | 0.839 |
| Intensive care delirium screening checklist (points) | 0 [0-1] | 0 [0-1] | 0.551 |
| Delirium, n/n assessed (%) | 5/43 (12%) | 3/8 (37%) | 0.099 |
| Medical research council (MRC) score (points) | 55 [42-60] | 32 [26-40] | **0.022** |
| ICU-acquired weakness (MRC < 48), n/n total (%) | 11/37 (30%) | 6/7 (86%) | **0.009** |
| Prophylactic non-invasive ventilation, n (%) | 21 (48%) | 5 (62%) | 0.703 |
| ***Sleep quantity*** |  |  |  |
| Duration of polysomnography recording (hours) | 16.7 [14.9-17.2] | 16.1 [14.5-17.9] | 0.919 |
| Sleep duration (hours) | 3.0 [1.3-4.5] | 2.2 [1.0-3.2] | 0.742 |
| Sleep efficiency (%) | 18 [7-24] | 12 [8-18] | 0.713 |
| Duration of light or atypical sleep (min) | 94 [46-187] | 95 [51-110] | 0.736 |
| Duration of deep sleep stage 3 (min) | 17 [0-93] | 20 [2-45] | 0.919 |
| Duration of REM sleep stage (min) | 0 [0-12] | 0 [0-0] | 0.163 |
| ***Sleep quality*** |  |  |  |
| Absence of deep sleep, n (%) | 26 (59%) | 7 (88%) | 0.232 |
| Absence of REM sleep, n (%) | 13 (30%) | 2 (25%) | 0.999 |
| Atypical sleep, n (%) | 8 (18%) | 2 (25%) | 0.642 |
| Pathological wakefulness, n (%) | 10 (23%) | 2 (25%) | 0.999 |
| Altered EEG reactivity at eye-opening test, n/n assessed (%) | 13/42 (31%) | 2/6 (33%) | 0.999 |
| ***Sleep fragmentation*** |  |  |  |
| Arousals and awakenings, events per hour of sleep | 27 [18-33] | 15 [11-21] | **0.039** |
| Average sound pressure level, decibels | 56 [54-61] | 58 [56-60] | 0.573 |
| Sound level events above 60 decibels, n events/hour | 25 [19-36}] | 38 [31-39] | 0.162 |

Values are given in median [25^th^-75^th^ percentiles] and compared using the non-parametric Fisher exact test for categorical variables and the Mann-Whitney test for continuous variables.

***Abbreviations:*** P_0.1_ = Negative airway pressure generated against occlusion during the first 0.1 s of spontaneous ventilation; REM sleep = Rapid eye movment sleep; ICU = Intensive care unit; EEG reactivity = Electroencephalographic reactivity;

*# Difficult or prolonged weaning refer to patients who were not extubated within the first 24 hours after the initial spontaneous breathing trial.*
